# Supplementary figures and images for: Population genomics of Plasmodium vivax in Panama to assess the risk of case importation on malaria elimination
Source: PLoS Negl Trop Dis. 2020 Dec 14;14(12):e0008962. doi: 10.1371/journal.pntd.0008962 (PMC7769613; doi:10.1371/journal.pntd.0008962)

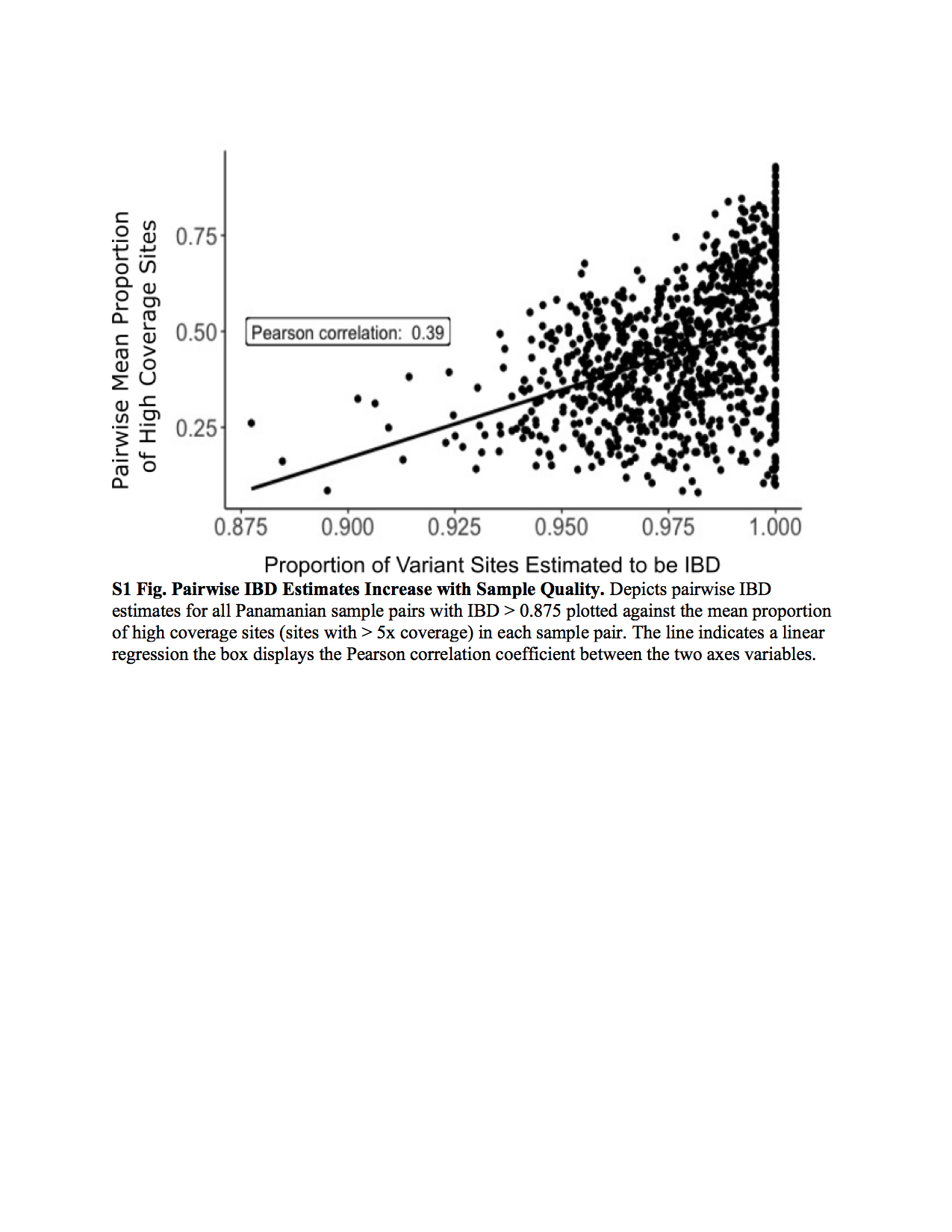

Supplement: S1 Fig — Depicts pairwise IBD estimates for all Panamanian sample pairs with IBD > 0.875 plotted against the mean proportion of high coverage sites (sites with > 5x coverage) in each sample pair. The line indicates a linear regression, the box displays the Pearson correlation coefficient between the two axes variables. (PNG) [file pntd.0008962.s001.png]

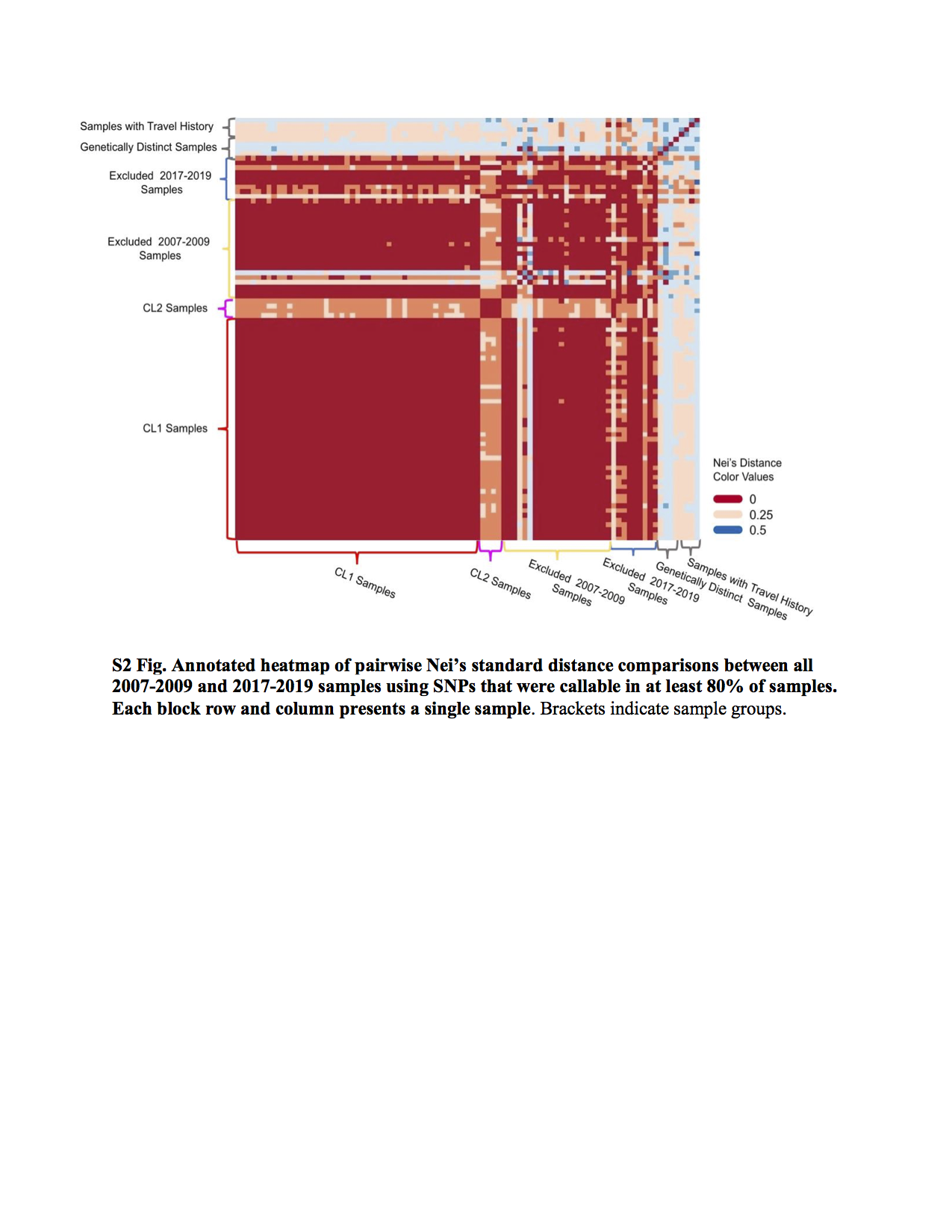

Supplement: S2 Fig — Each block row and column presents a single sample. Brackets indicate sample groups. (PNG) [file pntd.0008962.s002.png]

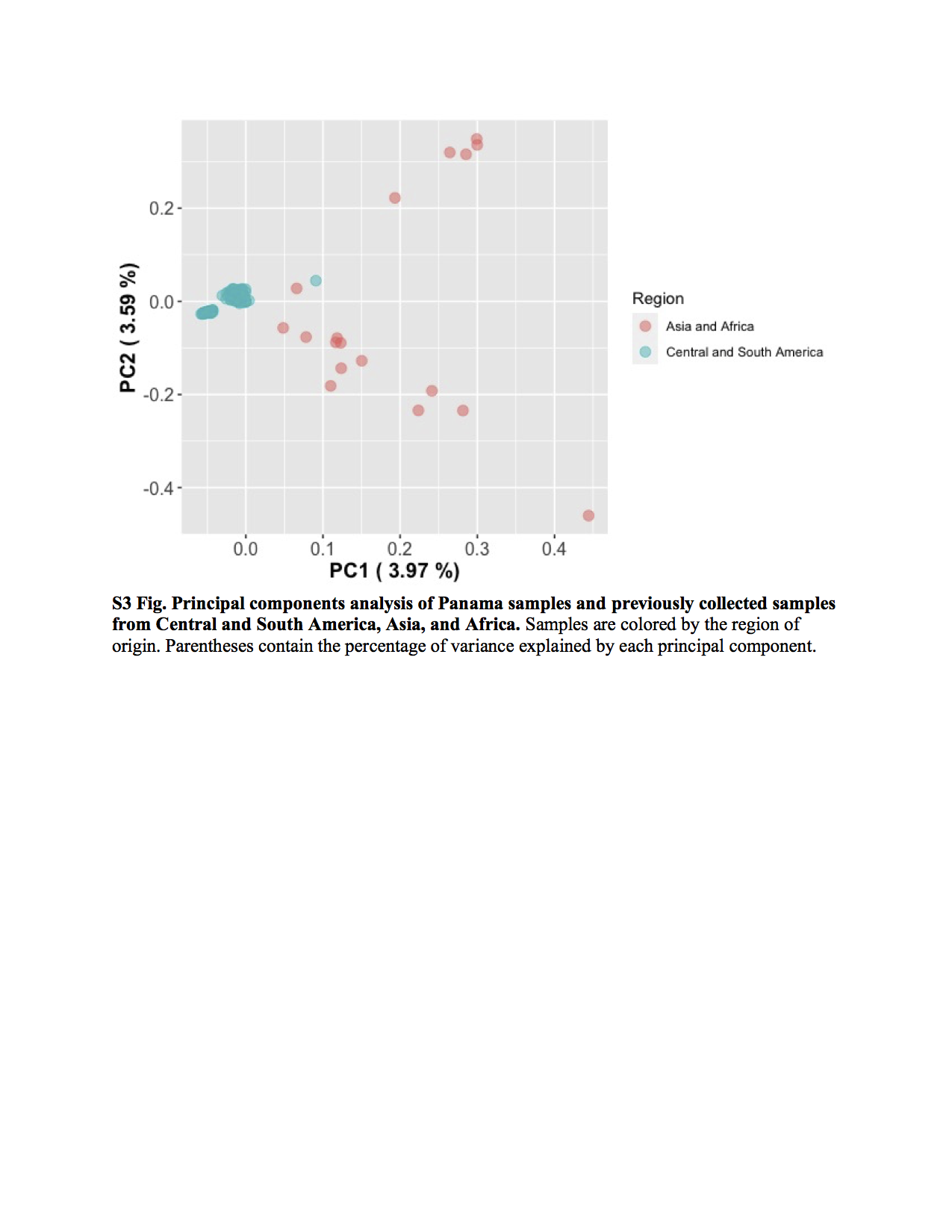

Supplement: S3 Fig — Samples are colored by the region of origin. Parentheses contain the percentage of variance explained by each principal component. (PNG) [file pntd.0008962.s003.png]

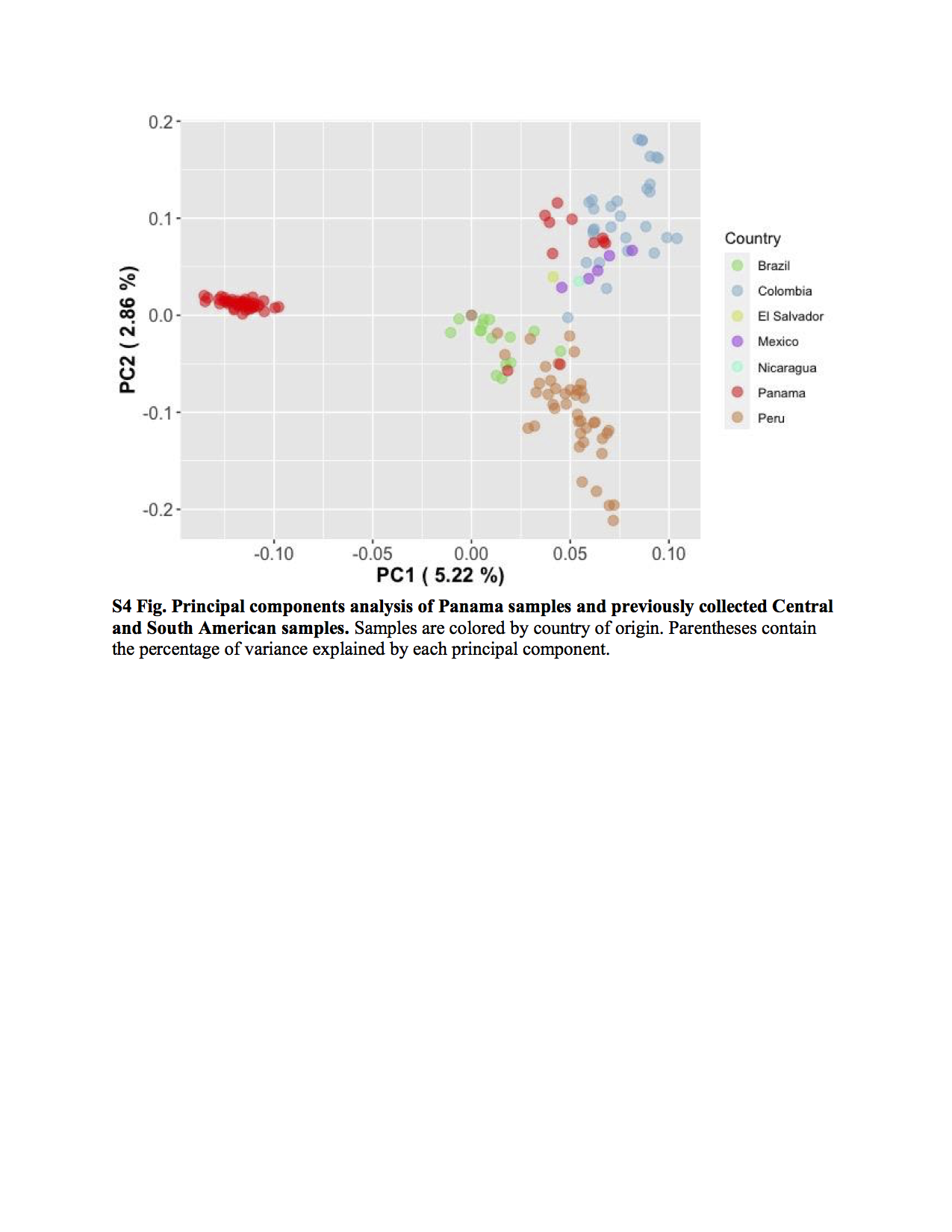

Supplement: S4 Fig — Samples are colored by country of origin. Parentheses contain the percentage of variance explained by each principal component. (PNG) [file pntd.0008962.s004.png]
