# Supplementary material for: Population genomics of Plasmodium vivax in Panama to assess the risk of case importation on malaria elimination
Source: PLoS Negl Trop Dis. 2020 Dec 14;14(12):e0008962. doi: 10.1371/journal.pntd.0008962 (PMC7769613; doi:10.1371/journal.pntd.0008962)
Supplement: Attachment — Submitted filename: PNTD-D-20-01524 review.docx [file pntd.0008962.s005.docx]

**Population Genomics of Plasmodium vivax in Panama to Assess the Risk of Case Importation on Malaria Elimination**

**General comments**

- This study provides a useful analysis, describing parasite population dynamics that may guide elimination strategies in Panama. The authors highlight a number of ways in the conclusions in which the data can inform policy - this could be elevated by briefly describing follow-up studies or programmatic actions to illustrate the translation of genomic data into real-time epidemiological application. Is genomic data ready for immediate inclusion in case investigation and response, or does it need to be paired with other data and surveillance in order to be actionable. If so, what are these things factors that need to be considered.

**Abstract and Author’s Summary**

- Line 35: “spanning the entire period of the collection (2007-2019)” and lines 56-57 “we collected 100 Panamanian P. vivax samples from two collection periods (2007- 2009 and 2017-2019).”
  - Can the authors clarify whether the samples were collected throughout the 2007-2019 period as indicated in the Abstract, or only in the periods 2007-2009 and 2017-2019 as indicated in the Author’s Summary? If the latter, it should probably be more specific in the introduction, as the reader might think this is continuous longitudinal sampling over a decade.
- Lines 53-55: “Understanding parasite transmission patterns and identifying imported cases is critical to help Panama and other countries succeed in their elimination efforts. Genomic epidemiology and population genomics can help provide information needed to inform malaria control policy.”
  - If word count allows, these sentences could elevate the intro of the paper by being a bit more specific in how the data could be used to guide elimination efforts or inform control policy. This could potentially have a Pv focus, which is why this research is unique and should be highlighted. E.g., how might “differentiation between imported infections or sustained local infection as the underlying cause of persistent P. vivax incidence” as mentioned in the introduction lead to policy decisions.

**Introduction**

- Lines 116-122: “We found the vast majority of P. vivax cases in Panama belong to a single highly related lineage that has persisted for at least a decade. Furthermore, we observed a second smaller clonal lineage concentrated near the Panamanian-Colombian border. We also found several samples that shared no relatedness with any other sample, which may represent either localized pockets of outbred P. vivax transmission or imported cases. These findings suggest the Panamanian P. vivax population has extremely low genetic diversity and is on the cusp of elimination”
  - These seem to be results, so should be left out of the introduction for now or moved to the results/conclusions section, if not already summarised there.

**Materials the methods**

- Should this section be moved up to come after the Introduction and before the Results? Check with PLoS formatting guidelines
- Lines 344 – 346 “We extracted DNA from the filter paper blood spots using the Chelex method as described (16) for samples obtained during 2007-2009 and with the Qiagen® DNA mini kit for samples obtained during 2017-2019.”
  - Would there be any expected impact on results due to different in extraction method? Is there a need to account for that potential in data analysis methods?
- Lines 409 -410: “to ascertain recent common ancestry among Panamanian and Colombian samples collected previously from the global P. vivax population study”.
  - Some sentences briefly giving some background to the global P.vivax population study would help the reader less familiar with the area.

**Results**

- Lines 156 – 157: “We observed that as the average proportion of high coverage sites for sample pairs 157 increased, pairwise IBD estimates correspondingly increased as well.”
  - Could the authors include briefly some of the factors that lead to low proportion of high coverage sites for samples? Is the distribution of the lower sample quality related to the sample collection periods, field collection/storage or extraction/sample processing methods that might introduce a systematic bias in the analysis?
- Lines 182-183: “the majority of excluded samples in both collection periods (17/21 and 4/16 for the 2007-2009 and 2017-2019 collection periods respectively) exhibited very low levels (0-1%) of genetic distance with CL1 samples. Seven of the 2017-2019 samples exhibited 0-1% genetic distance with CL1 also showed 0-1% 186 genetic distance with CL2, making the identity of these samples uncertain.”
  - This means that the majority of the excluded samples e.g., of the 21 excluded samples, 17 had low levels of genetic distance from CL1, but only 4 of the 16 excluded samples in the later collection period had low genetic distance? However, the next sentence indicates that 7 samples from the latter collection period had low CL1 genetic distance. Does this mean more specifically that the majority of the samples collected in the earlier period were those that showed low genetic distance, but less than half of those in the latter collection period did? Could the authors clarify? Does this indicate anything about bias introduced by sample collection or processing, as per comment above?

**Discussion**

- Line 261: “variable malaria transmission”
  - Do the authors mean to say spatially heterogeneous or focal transmission (as opposed to temporally variable)?
