# Supplementary material for: Population genomics of Plasmodium vivax in Panama to assess the risk of case importation on malaria elimination
Source: PLoS Negl Trop Dis. 2020 Dec 14;14(12):e0008962. doi: 10.1371/journal.pntd.0008962 (PMC7769613; doi:10.1371/journal.pntd.0008962)
Supplement: Attachment — Submitted filename: Response Letter.docx [file pntd.0008962.s006.docx]

We thank the reviewers for their thoughtful comments. We have revised the manuscript significantly in response to the points raised, and believe the manuscript is substantially improved. Below we respond (in bold) to specific comments.

Reviewer's Responses to Questions

Key Review Criteria Required for Acceptance?

As you describe the new analyses required for acceptance, please consider the following:

Methods

-Are the objectives of the study clearly articulated with a clear testable hypothesis stated?

-Is the study design appropriate to address the stated objectives?

-Is the population clearly described and appropriate for the hypothesis being tested?

-Is the sample size sufficient to ensure adequate power to address the hypothesis being tested?

-Were correct statistical analysis used to support conclusions?

-Are there concerns about ethical or regulatory requirements being met?

Reviewer #1: (No Response)

Reviewer #2: The methods are well-suited for the questions addressed and the analyses well-conducted.

Reviewer #3: -Are the objectives of the study clearly articulated with a clear testable hypothesis stated? Yes

-Is the study design appropriate to address the stated objectives? Yes

-Is the population clearly described and appropriate for the hypothesis being tested? Yes

-Is the sample size sufficient to ensure adequate power to address the hypothesis being tested? Unable to assess

-Were correct statistical analysis used to support conclusions? Yes

-Are there concerns about ethical or regulatory requirements being met? No

Abstract and Author’s Summary

Line 35: “spanning the entire period of the collection (2007-2019)” and lines 56-57 “we collected 100 Panamanian P. vivax samples from two collection periods (2007- 2009 and 2017-2019).”

o	Can the authors clarify whether the samples were collected throughout the 2007-2019 period as indicated in the Abstract, or only in the periods 2007-2009 and 2017-2019 as indicated in the Author’s Summary? If the latter, it should probably be more specific in the introduction, as the reader might think this is continuous longitudinal sampling over a decade.

**We have added language to clarify that samples were collected in two different sampling periods on line 28-31: “We used selective whole genome amplification to sequence 59 *P. vivax* samples from Panama. The *P. vivax* samples were collected from two periods (2007-2009 and 2017-2019) to study the population structure and transmission dynamics of the parasite.”**

Methods

Lines 344 – 346 “We extracted DNA from the filter paper blood spots using the Chelex method as described (16) for samples obtained during 2007-2009 and with the Qiagen® DNA mini kit for samples obtained during 2017-2019.”

o	Would there be any expected impact on results due to differences in extraction method? Is there a need to account for that potential in data analysis methods?

**We thank the reviewer for pointing out this consideration. We acknowledge that this factor could potentially introduce stratification into the analyses via, for example, differences in sequencing coverage. We note that we observed CL1 in both sample batches, and use a strict set of SNPs for analysis that kept SNPs only with 5 < DP < 30 for analysis to limit any biases resulting from systematic differences in read depth coverage. We describe this assessment of analysis stratification risk in the Discussion (530-538):**

**“Factors such as differences in DNA extraction techniques used for the two sample collection periods or length of storage of the samples could have affected DNA yield and/or molecular weight, impacting SWGA. Some samples may have had lower coverage due to lower parasitemia. Dissimilarities in coverage between the early and late sample batches could be due to technical factors such as different flowcell loading. We did not find an association between coverage and geographic location. Low sequencing coverage for some samples may have limited the sensitivity of the F_ws_ statistic to detect polyclonal infections. However we used a filtered dataset of SNPs for F_ws_ calculation that had a minimum of coverage of 5x and a maximum coverage of 30x to minimize bias from coverage.”**

Lines 409 -410: “to ascertain recent common ancestry among Panamanian and Colombian samples collected previously from the global P. vivax population study”.

o	Some sentences briefly giving some background to the global P. vivax population study would help the reader less familiar with the area.

**We have added text to contextualize this study as suggested on lines 493-498: “Several previous studies found that the Central American and South American *P. vivax* populations are distinct from each other** [**(20,30,38)**](https://www.zotero.org/google-docs/?kCjY89)**. A previous work suggests this population structure is due to multiple founding events after likely European introduction (39). This population structure could also be due genetic drift since founding. However, the Panamanian *P. vivax* population has been through too severe of a bottleneck to help clarify historical causes of this population structure with the present data.”**

--------------------

Results

-Does the analysis presented match the analysis plan?

-Are the results clearly and completely presented?

-Are the figures (Tables, Images) of sufficient quality for clarity?

Reviewer #1: (No Response)

Reviewer #2: Fine, although I would have liked to see a display of the pairwise IBD.

**We have revised Figure 2 to include a figure of pairwise IBD in panel 2B in place of the original Figure 2B. We have moved the original figure 2B to the supplemental information section.**

Reviewer #3:

-Are the results clearly and completely presented? Some clarifications needed regarding proportion of samples excluded with low genetic distance from CL1.

**We added the following language on lines 398-401 to clarify this: “The majority of excluded samples across both collection periods (17/21 and 4/16 for the 2007-2009 and 2017-2019 collection periods respectively; a total of 21/37 samples) exhibited very low levels (0-1%) of genetic distance with the CL1 samples and higher genetic distance (0.2-0.25) with the CL2 samples (S2 Fig).” and lines 405-407: “Two samples from the 2017-2019 collection period share very low genetic distance (0-0.1) with the CL2 samples, and higher genetic distance (0.15-0.25) from the CL1 samples.”**

Results

Lines 156 – 157: “We observed that as the average proportion of high coverage sites for sample pairs 157 increased, pairwise IBD estimates correspondingly increased as well.”

o	Could the authors include briefly some of the factors that lead to low proportion of high coverage sites for samples? Is the distribution of the lower sample quality related to the sample collection periods, field collection/storage or extraction/sample processing methods that might introduce a systematic bias in the analysis?

**The causes of low coverage are not certain, but these results are not atypical for *Plasmodium* samples sequenced via SWGA. We do not believe that low coverage is related to the different time periods of sample collection or that it would necessarily result in systematic bias. The samples were sequenced in different sequencing runs and therefore technical factors such as different flowcell loading may explain coverage differences to some extent. We also note that we observe CL1 in both sets of samples.**

**We have added language to the Discussion expanding on reasons for low coverage (lines 530-548). The excerpt is referenced previously in this letter.**

Lines 182-183: “the majority of excluded samples in both collection periods (17/21 and 4/16 for the 2007-2009 and 2017-2019 collection periods respectively) exhibited very low levels (0-1%) of genetic distance with CL1 samples. Seven of the 2017-2019 samples exhibited 0-1% genetic distance with CL1 also showed 0-1% genetic distance with CL2, making the identity of these samples uncertain.”

o	This means that the majority of the excluded samples e.g., of the 21 excluded samples, 17 had low levels of genetic distance from CL1, but only 4 of the 16 excluded samples in the later collection period had low genetic distance? However, the next sentence indicates that 7 samples from the latter collection period had low CL1 genetic distance. Does this mean more specifically that the majority of the samples collected in the earlier period were those that showed low genetic distance, but less than half of those in the latter collection period did? Could the authors clarify? Does this indicate anything about bias introduced by sample collection or processing, as per comment above?

**The 7 samples that share no genetic distance with both CL1 and CL2 are due to low coverage and high proportion of missing calls which we note in the discussion. We expand upon the uncertain identity of the 7 samples in the results on lines 401-403: “Seven samples in the 2017-2019 collection period all had a high proportion of missing calls for even these 264 SNPs, making distance measures uninformative.”**

**We expand upon the classification of our excluded samples in the discussion on lines 538-545: “We also used this a small set of 264 SNPs set to that were called in ~80% of samples to calculate Nei’s standard genetic distance to determine if the excluded samples were genetically distant from CL1 or CL2. We found that the majority of the excluded samples were genetically similar to CL1 (S2 Fig). This result indicates that our assessment of relatedness within the Panamanian *P. vivax* population is not biased by parasitemia or other factors that could have affected sequencing success. This finding also suggests that we did not miss additional genetically distinct circulating Panamanian *P. viva*x strains and thus did not bias our analysis by excluding these samples.”**

--------------------

Conclusions

-Are the conclusions supported by the data presented?

-Are the limitations of analysis clearly described?

-Do the authors discuss how these data can be helpful to advance our understanding of the topic under study?

-Is public health relevance addressed?

Reviewer #1: A recent study using microsatellites (<https://journals.plos.org/plosntds/article?id=10.1371/journal.pntd.0008072>) and not so recent one (<https://journals.plos.org/plosntds/article?id=10.1371/journal.pntd.0008072>), as well as WGS data (<https://www.nature.com/articles/ng.3588>) point to a P. vivax population in Central America and Mexico that it is clearly separated from South America. I am surprised these studies as not mentioned and discussed in light of the new data.

Further, on lines 241-246, I am puzzled that the authors decided to discuss similar patterns observed for P. falciparum, but not P. vivax. Clonal or near-clonal P. vivax populations have been described before, e.g. here <https://www.nature.com/articles/s41467-018-04965-4>. While P. falciparum can be discussed, the evidence we have on P. vivax should not be omitted. Overall this part could be referenced better.

**We have incorporated the studies you mention here and added language to the manuscript to better place the study results in context with previous work on lines 480-498: “Similar reductions in clonal diversity of *P. vivax* populations have been observed elsewhere. For example, a study investigating the relationship between P. vivax transmission intensity and genetic diversity in Malaysia** [**(31)**](https://www.zotero.org/google-docs/?gyN7aL) **documented that when there is a decline in parasite transmission, there is an increase in the clonal composition of the population. Several studies of *P. falciparum* genetic diversity and transmission intensity from Senegal** [**(32)**](https://www.zotero.org/google-docs/?7rdJ9a)**, Thailand** [**(33,34)**](https://www.zotero.org/google-docs/?Ji4CSM)**, and Colombia** [**(34) have**](https://www.zotero.org/google-docs/?7EVvxb) **also noted the same relationship. Furthermore, there is evidence of persistence and transmission of *P. vivax* clonal lineages in Malaysia** [**(31)**](https://www.zotero.org/google-docs/?VathUq)**, and *P. falciparum* clonal lineages in Colombia** [**(35)**](https://www.zotero.org/google-docs/?D1mSVw)**, Ecuador** [**(36)**](https://www.zotero.org/google-docs/?IkqbHN)**, and Haiti** [**(37)**](https://www.zotero.org/google-docs/?NFX5ZS)**. Our study demonstrates a similar relationship in Panama between low transmission and extremely low genetic diversity of the *P. vivax* population. Almost all CL1 samples (46/47) share IBD > 0.95 with at least one other CL1 sample, suggesting a substantial fraction of this population is clonal. Several previous studies found that the Central and South American P. vivax populations are distinct from each other** [**(20,30,38)**](https://www.zotero.org/google-docs/?kCjY89)**. A previous study suggested this population structure is due to multiple founding events after likely European introduction** [**(39).**](https://www.zotero.org/google-docs/?sfAui9) **This structure could also be due to genetic drift since founding. However, the Panamanian *P. vivax* population has been through too severe of a bottleneck to help clarify historical causes of this population structure with the present data.”**

Reviewer #2: The discussion of the ability of genome sequence data to support inference of imported cases should be expanded (see below).

**We address this comment later in the letter.**

Reviewer #3: -Are the conclusions supported by the data presented? Yes

-Are the limitations of analysis clearly described? Some description of the factors that might lead to the limitations highlighted by the authors would be useful.

**We have expanded on these limitations in the discussion, lines 530-538:**

**“Factors such as differences in DNA extraction techniques used for the two sample collection periods or length of storage of the samples could have affected DNA yield and/or molecular weight, impacting SWGA. Some samples may have had lower coverage due to lower parasitemia. Dissimilarities in coverage between the early and late sample batches could be due to technical factors such as different flowcell loading. We did not find an association between coverage and geographic location. Low sequencing coverage for some samples may have limited the sensitivity of the F_ws_ statistic to detect polyclonal infections. However we used a filtered dataset of SNPs for F_ws_ calculation that had a minimum of coverage of 5x and a maximum coverage of 30x to minimize bias from coverage.”**

-Do the authors discuss how these data can be helpful to advance our understanding of the topic under study? Yes

-Is public health relevance addressed? Yes

Line 261: “variable malaria transmission”

o	Do the authors mean to say spatially heterogeneous or focal transmission (as opposed to temporally variable)?

**We have revised the language here to better place the result in context with previous work on lines 504-504: “Prior work shows that low transmission can lead to an increase in clonal population structure”.**

--------------------

Editorial and Data Presentation Modifications?<br/>

Use this section for editorial suggestions as well as relatively minor modifications of existing data that would enhance clarity. If the only modifications needed are minor and/or editorial, you may wish to recommend “Minor Revision” or “Accept”.

Reviewer #1: (No Response)

Reviewer #2: (No Response)

Reviewer #3: Abstract and Author’s Summary

Lines 53-55: “Understanding parasite transmission patterns and identifying imported cases is critical to help Panama and other countries succeed in their elimination efforts. Genomic epidemiology and population genomics can help provide information needed to inform malaria control policy.”

o	If word count allows, these sentences could elevate the intro of the paper by being a bit more specific in how the data could be used to guide elimination efforts or inform control policy. This could potentially have a Pv focus, which is why this research is unique and should be highlighted. E.g., how might “differentiation between imported infections or sustained local infection as the underlying cause of persistent P. vivax incidence” as mentioned in the introduction lead to policy decisions.

**We thank the reviewer for the comment and have reworded this section of the introduction and expanded upon the specific applications of genomic data for policy recommendations. On lines 140-143 we have added “ To inform effective malaria elimination strategies in Panama, it is critical to understand how the parasite moves throughout the country, uncover pockets of focalized transmission, and differentiate between sustained local infection vs case importation as the reason for disease persistence.”**

Introduction

Lines 116-122: “We found the vast majority of P. vivax cases in Panama belong to a single highly related lineage that has persisted for at least a decade. Furthermore, we observed a second smaller clonal lineage concentrated near the Panamanian-Colombian border. We also found several samples that shared no relatedness with any other sample, which may represent either localized pockets of outbred P. vivax transmission or imported cases. These findings suggest the Panamanian P. vivax population has extremely low genetic diversity and is on the cusp of elimination”

o	These seem to be results, so should be left out of the introduction for now or moved to the results/conclusions section, if not already summarised there.

**We have modified this section to less explicitly describe the results on lines 161-164 to simply say “Revealing these patterns of relatedness among parasite infections can help inform best strategies for targeting interventions or case investigation methods to increase the likelihood of successful elimination. We discuss these findings and their implications for ongoing elimination efforts of *P. vivax* in Panama.”**

Materials and Methods

Should this section be moved up to come after the Introduction and before the Results? Check with PLoS formatting guidelines

**We have moved up this section.**

Discussion

Line 261: “variable malaria transmission”

o	Do the authors mean to say spatially heterogeneous or focal transmission (as opposed to temporally variable)?

**We have received the language here to better place the result in context with previous work on line 504-505: “Prior work shows that low transmission can lead to an increase in clonal population structure.”**

--------------------

Summary and General Comments

Use this section to provide overall comments, discuss strengths/weaknesses of the study, novelty, significance, general execution and scholarship. You may also include additional comments for the author, including concerns about dual publication, research ethics, or publication ethics. If requesting major revision, please articulate the new experiments that are needed.

Reviewer #1: This study presents interesting data, showing very strong clonality of P. vivax in Panama, with some lineages persisting for over 10 years. It is mostly well written and I do not have any major concerns, but there are a few points that are misleading and need to be corrected.

Further, I feel the discussion misses the opportunity to better discuss the new data in light of previous studies pointing to a genetically unique Central American P. vivax population, and similar clonal population structure from other parts of the world (I wonder why P. falciparum is discussed instead).

Minor comments

Line 41: It is difficult to understand what is meant by “The small number of genetically unique …”. It would be clearer to mention CL1 and CL2 in lines 34-37, and then on line 41 to clarify that CL2 is referred to.

**We have added clarifying language on lines 33-45: “We explored patterns of recent common ancestry among the samples and observed that a single highly genetically related lineage (Termed CL1) was dominant among the samples (47 out of 59 samples with good sequencing coverage), spanning the entire period of the collection (2007-2019) and all regions of the country. We also found a second, smaller clonal lineage (Termed CL2) of four parasites collected between 2017 and 2019. To explore the regional context of Panamanian *P. vivax* we conducted principal components analysis and constructed a neighbor-joining tree using these samples and samples collected worldwide from a previous study. Three of the four samples with travel history clustered with samples collected from their suspected country of origin (consistent with importation), while one appears to have been a result of local transmission. The small number of Panamanian *P. vivax* samples not belonging to either CL1 or CL2 clustered with samples collected from Colombia, suggesting they represent the genetically similar ancestral *P. vivax* population in Panama or were recently imported from Colombia or elsewhere.”**

Line 72: I believe it is now established that P. ovale wallikeri and P. ovale curtisi are 2 different species.

**We have changed the language of this section to reflect this on lines 103-105: “*Plasmodium falciparum,* the most virulent of the six *Plasmodium* species that infect humans (*P. falciparum*, *P. vivax, P. malariae, P. ovale wallikeri, P. ovale curtisi, and P. knowlesi*) causes the majority of these cases.”**

Line 77-78: “However, recent studies suggest this parasite species causes a significant global health burden (1,2).”

Reference 2 is from 2007, I would not call that ‘recent’

**We have removed the word.**

Line 79: P. ovale also forms hypnozoites, thus they are not unique to Pv

**We have deleted this statement and instead say on line 110: “The *P. vivax* cycle includes a dormant liver “hypnozoite” stage.”**

Line 79: It is very rare that Pv relapses years after the initial infection, most relapses occur much earlier. Please provide a more realistic statement.

**We have added the statement of “weeks-to-months” on line 111.**

Define IBD at first use

**We have added a definition of IBD in the Results section when it is first mentioned on lines 333-335: “ IBD measures the proportion of the genome between two individuals that was inherited from a recent common ancestor. Pairwise IBD values closer to 100% indicate very recent common ancestry.”**

Define VCF at first use. I also wonder why the authors do not use the term ‘data set’ or similar instead of repeatedly refer to ‘VCF’ (i.e. the file format).

**We have defined VCF and changed language to ‘dataset’ in the Results and Discussion sections.**

Lines 178-188: When the analysis was restricted to 264 SNPs, did any results change for the samples already analyzed with the WGS data? Did some samples no longer cluster with CL1 or CL2? It is only described how the additional samples cluster, but not whether any results had changed if all samples had been typed with only 264 SNPs.

**When we restrict analysis to these 264 SNPs and include all of the samples, it does not change the results. We note that even when we restrict analysis to these 264 SNPS we will see differences in genetic distance between CL1 and CL2 samples and the consensus sequence. We have updated the text to reflect this point on lines 407-408: “The previously observed sample clustering patterns did not change when conducting the analysis with the 264 SNPs .”**

Line 212: The title “Genomic data both support and refute travel history data” is misleading and leaves the reader wonder what is going on. Stating “Genomic data matches travel history in three out of four cases” would be much clearer.

**We have made this change.**

Line 242: Reference 30 is a study on P. vivax, not P. falciparum.

**An issue arose with our bibliographic software, resulting in the wrong reference number. We have fixed this for resubmission.**

Lines 310-311: “While case importation remains a threat, the lack of evidence of outcrossing suggests it may not be sufficient to prevent elimination under present circumstances.”

This sentence is confusing. Should “outcrossing” be replaced with “onward transmission”? I don’t see why outcrossing per se is a requirement for imported infections to be a thread for elimination.

**We agree with this suggested language adds clarity and we have added it on line 594-594: “While case importation remains a threat, the lack of evidence of ongoing transmission outcrossing suggests it may not be sufficient to prevent elimination under present circumstances.”**

Line 313: It appears a word is missing before ‘reversal’, likely it should read ‘prevent reversal’.

**We have made this correction.**

Reviewer #2: The study by Buyon et al. describes 59 genome sequences from Panama and their analyses with respect to the global and continental diversity of P. vivax. The data are interesting and the analyses, for the most part, well conducted and convincing. I have some minor comments and suggestions on the presentation of the analyses, but the manuscript is overall well written and interesting. My main comment is to expand the discussion of how genetic information can (or cannot) inform on case importation based on the region genetic diversity and the specificities of P. vivax biology.

Main comment:

I think the authors missed an opportunity to discuss the ability to identify imported cases using genetic data (which would better reflect the title of the paper). Based on their data and analyses, it is not clear that genomic information provides sufficient information to rigorously identify the origin of a parasite: the differences between continents is clear and convincing but it is not obvious that one can discriminate with high confidence clones from Brazil vs. Peru, or Mexico vs. Nicaragua. Maybe the authors can provide some statistical support for such analyses and discuss the limitations for malaria control (e.g. identifying robustly imported cases and/or the origin to these cases). The high clonality of Panama is clearly an advantage in this case and this could be also discussed (as the prevalence goes down, it might be easier to identify imported cases). Finally, the role of dormant P. vivax parasites should be at least mentioned as a potential complication when trying to reconcile recent travel history and parasite genetic diversity

**In this case we were not trying to attribute samples to specific countries, but to resolve local vs imported cases. The former application would benefit from a much deeper training dataset of samples from potential countries of origin than what we used for the tree and PCA. We acknowledge country of origin attribution as an application to be further developed. In the case of Panama where onward transmission of imported infections appears to be rare, if genetic data indicate an infection is imported, travel history data probably could identify the country of origin more easily than a genetic attribution analysis. We also agree that dormant *P. vivax* could complicate such analysis. We have added text to the discussion addressing this point on lines 579-583: “ Relapsing *P. vivax* infections resulting from dormant hypnozoites could complicate reconciliation of travel history with genomic data if infections were acquired months previously. “Further development of tools using a benchmarked set of markers, such as SNP barcodes**[**(41,42)**](https://www.zotero.org/google-docs/?WdfGef) **, for each *P. vivax* endemic country would help to identify parasite country of origin solely using genomic data.”**

Minor comments and editorial suggestions:

Complexity of infection. The sequence coverage for many samples is very low (especially for the 2017-2019 samples). This limits the ability of the authors to detect polyclonal infections (if any) and this limitation should be at least noted. The COI (or lack thereof) could also be used in the discussion as further support for the statement that the P. vivax population in Panama has low genetic diversity and is highly clonal.

**With regards to COI, while overall coverage for some samples was low, we restricted our input SNPs for F_ws_ calculation to those that had a coverage depth of at least 5X, as recommended in the original Manske, M., Miotto, O. et al 2012 paper. We thought that COI would be redundant to F_ws_ in this instance, and that by carefully filtering the input SNPs for F_ws_ calculation we would avoid missing polyclonal infections composed of partially related parasite lineages. Additionally the high pairwise IBD between samples would reduce our ability to accurately measure COI through heterozygosity since polyclonal infections could be partially IBD. We do agree that we should note that low coverage leading to over estimation of F_ws_ is a limitation and have added that to the manuscript on lines 530-538. The excerpt is referenced previously in this letter.**

Figure 4. IBD analysis. Instead of Fig2B (that could be moved to supplemental), I would have like to see the pairwise IBD among samples from Panama (e.g., a heatmap of IBD), with maybe the Colombian samples to provide a comparison point. This would strengthen the point the authors made and emphasize the uniqueness of the P. vivax population in Panama. It would be great if the authors could use the same colors and symbols for A and B

**We have updated Figure 2 to include a heatmap of pairwise IBD. We wanted to explicitly highlight the samples with travel history on the PCA and felt that adding a symbol would be too subtle. The figures and colors are the same but we have fixed the resolution so that the color presentation is clearer.**

Line 29: emphasizing the “96 samples” in the abstract is a little misleading and the authors should replace by the 59 actually successfully sequenced and analyzed.

**We note this and have changed the wording on lines 28-31: “We used selective whole genome amplification to sequence 59 *P. vivax* samples from Panama. The *P. vivax* samples were collected from two periods (2007-2009 and 2017-2019) to study the population structure and transmission dynamics of the parasite.”**

Line 139: please indicate the number of Colombian samples included

**We have added this number (30) on line 290.**

Line 171-174: Duplicated information. Remove the first sentence of the paragraph?

**We have changed the wording.**

Line 198: replace “zero” by “little”

**We have changed the wording.**

Line 234: I am not sure to understand correctly the statement of “extremely low clonal diversity”… Replace by “extremely low genetic diversity” or “high clonality”

**We have changed the wording to ‘extremely high clonality’.**

David Serre

Reviewer #3: This study provides a useful analysis, describing parasite population dynamics that may guide elimination strategies in Panama. The authors highlight a number of ways in the conclusions in which the data can inform policy - this could be elevated by briefly describing follow-up studies or programmatic actions to illustrate the translation of genomic data into real-time epidemiological application. Is genomic data ready for immediate inclusion in case investigation and response, or does it need to be paired with other data and surveillance in order to be actionable. If so, what are these things factors that need to be considered.

**We thank the reviewer for the helpful suggestion to better situate these results in context with policy. We note in the paper several ways to incorporate genomic data into policy, and we added some language expanding the exploration of translating genomic data into policy. We do note in the paper that Genomic data can and should be paired with travel history data to enhance identification of imported cases. We also note that ongoing surveillance is critical and genomic surveillance will be paired and added some language (lines 595-599) to make this more explicit : “The ability to use genomics to identify imported cases will be improved by collecting genomic data from other countries in the region and making them freely available to use as a population genomics reference to identify recent common ancestry**.”
